# Supplementary material for: Borrelia burgdorferi Migration Assays for Evaluation of Chemoattractants in Tick Saliva
Source: Pathogens. 2022 May 1;11(5):530. doi: 10.3390/pathogens11050530 (PMC9147933; doi:10.3390/pathogens11050530)
Supplement: Supplementary file 1 [file pathogens-11-00530-s001.zip › pathogens-1692123-supplementary.pdf]

Supplemental Table S1. Summary of Results Using the U-tube Assay.

Matrix	Matrix Volume (µl)	Cells	[Cell] (x10^7/ml)	Inocula: CA or control	[CA]	Incubation Environment	Incubation Time (Hours)	Final Cell Count
0.1% agarose in BSK-II	450	in vitro Bb	2.1	D-GlcNAc	100mM	34˚C trigas	96	0
0.1% agarose in BSK-II	450	in vitro Bb	2.1	PBS		34˚C trigas	96	0
0.1% agarose in BSK-II	300	in vitro Bb	2.3	D-GlcNAc	100mM	34˚C trigas	168	0
0.1% agarose in BSK-II	300	in vitro Bb	2.3	PBS		34˚C trigas	168	0
0.1% agarose in BSK-II	300	in vitro Bb	2	D-GlcNAc	500mM	34˚C trigas	120	0
0.1% agarose in BSK-II	300	in vitro Bb	2	PBS		34˚C trigas	120	0
0.1% agarose in BSK-II	300	in vitro Bb	1.8	tick saliva	1%	34˚C trigas	72	0
0.1% agarose in BSK-II	300	in vitro Bb	1.8	PBS		34˚C trigas	72	0
0.1% agarose in PBS	300	in vitro Bb	2.8	chitobiose	10mM	34˚C trigas	19	0
0.1% agarose in PBS	300	in vitro Bb	2.8	PBS		34˚C trigas	19	0
0.1% agarose in PBS	300	in vitro Bb	2.4	chitobiose	10mM	34˚C trigas	24	0
0.1% agarose in PBS	300	in vitro Bb	2.4	PBS		34˚C trigas	24	0
0.1% agarose in PBS	300	in vitro Bb	2.4	chitobiose	10mM	34˚C trigas	24	2/cs
0.1% agarose in PBS	300	in vitro Bb	2.4	PBS		34˚C trigas	24	0
0.1% agarose in low sugar BSK-II	300	in vitro Bb	2	chitobiose	10mM	34˚C trigas	45	0
0.1% agarose in low sugar BSK-II	300	in vitro Bb	2	low sugar BSK-II		34˚C trigas	45	0
0.1% agarose in low sugar BSK-II	300	Host-adapted Bb	3	chitobiose	10mM	34˚C trigas	72	0
0.1% agarose in low sugar BSK-II	300	Host-adapted Bb	3	D-GlcNAc	100mM	34˚C trigas	72	0

0.1% agarose in low sugar BSK-II	300	Host-adapted Bb	3	low sugar BSK-II		34˚C trigas	72	0
0.1% agarose in low sugar BSK-II	300	Host-adapted Bb	3	tick saliva	5%	34˚C trigas	72	0
0.1% agarose in low sugar BSK-II	300	Host-adapted Bb	3	chitobiose	10mM	34˚C trigas	72	0
0.1% agarose in low sugar BSK-II	300	Host-adapted Bb	3	low sugar BSK-II		34˚C trigas	72	0
4% gelatin in low sugar BSK-II	300	Host-adapted Bb	1.8	chitobiose in low sugar BSK-II	10mM	23˚C	48	0
